# The Anti-depression Effect of Angelicae Sinensis Radix Is Related to the Pharmacological Activity of Modulating the Hematological Anomalies

**DOI:** 10.3389/fphar.2019.00192

**Published:** 2019-03-06

**Authors:** Wenxia Gong, Shiwei Zhu, Congcong Chen, Qicai Yin, Xiao Li, Guanhua Du, Yuzhi Zhou, Xuemei Qin

**Affiliations:** ^1^Modern Research Center for Traditional Chinese Medicine, Shanxi University, Taiyuan, China; ^2^Institute of Materia Medica, Chinese Academy of Medical Sciences and Peking Union Medical College, Beijing, China

**Keywords:** Angelicae Sinensis Radix, anti-depression, anemia, metabonomics, hypoxia, sphingolipid metabolism

## Abstract

Angelicae Sinensis Radix (AS), a well-known herb in traditional Chinese medicine (TCM), has been wildly used for replenishing the blood and promoting circulation, in Asia for thousands of years. It has been confirmed that AS also possesses the pharmacological activity of anti-depression. At the same time, recent studies suggested that depression is associated with anemia, and depression could be ameliorated via modulating the blood system. However, it is still unknown whether the anti-depression effect of AS is related to its pharmacological activity of modulating the blood system. In the current study, hematological examination and metabonomic techniques were performed to explore potential anti-depression mechanisms of AS, related to the function of modulating the blood system in a chronic unpredictable mild stress (CUMS) model. The results demonstrated that AS could significantly improve CUMS-induced depressive symptom, hematological anomalies, and hypoxia symptoms. The analysis of metabonomics demonstrated that 26 potential biomarkers in depression could be regulated by the administration of AS. Among them, eight biomarkers participate in the metabolic pathways of amino acid and sphingolipid, and energy metabolism could also be regulated in an anemia model through the administration of AS, as reported in previous literatures. Further results proved that AS modulated energy metabolism in depression through the inhibition of the expression of pyruvate dehydrogenase lipoamide kinase isozyme 1 (PDK-1) and lactate dehydrogenase A (LDHA). These results suggested that the modulation of the blood system was involved in the anti-depression effect of AS. The mechanism may be associated with the promotion of the body’s energy metabolism, the stabilization of cell membranes, the promotion of serum protein synthesis, and the enhancement of immunity.

## Introduction

Angelicae Sinensis Radix (AS), also known as Danggui in China, has been used widely in Asia for thousands of years because of its pharmaceutical effects on gynecological, cardiovascular and cerebrovascular diseases. In recent years, the pharmacological activities of this herb in mental diseases, has attracted attention. Previous reports have demonstrated that AS ethanol extract could attenuate CUMS-induced depressive symptoms by mediating the brain derived neurotrophic factor (BDNF) signaling pathway (Shen et al., 2016). AS water extracts also significantly decreased the immobility time of mice in both a tail suspension test (TST) and a forced swimming test (FST) through improving the content of monoamine neurotransmitters and neurotrophic factor in the hippocampus (Liu et al., 2017). This evidence proved that AS possessed an exact antidepressant effect. On the other hand, AS is frequently used in classical anti-depressant formulas, such as Xiaoyao San (Wang et al., 2018) and Danggui Shaoyao San (Xu et al., 2011; Zhou et al., 2015). In addition, several reports demonstrated that some main constituents of AS indeed possessed anti-depression activity. For example, ferulic acid (FA) induced anti-depression via modulating the serotonergic system, regulating the HPA axis, and increasing ghrelin (Zhang et al., 2011; Zeni et al., 2012). Vanillic acid reversed the dysfunction of cognitive and memory induced oxidative stress via reducing AChE, TNF-α, and corticosterone (Singh et al., 2015). Butylphthalide improved lipopolysaccharide-induced depressive-like behavior in rats by regulating Nrf2 and NF-κB pathways (Yang et al., 2018). Despite the discovery of the anti-depression effect of AS, the underlying mechanism has not been investigated yet.

In recent years, several related studies suggested that depression is associated with anemia (Pamuk et al., 2016; Vulser et al., 2016). On the one hand, malnutrition in depressed patients may contribute to anemia (Mitrache et al., 2001; Quirk et al., 2013). On the other hand, anemia directly affects brain function and can result in cognitive impairment (Pickett et al., 1999), which contributes to the development of depression. Poorer physical performance associated with anemia has detrimental consequences on life quality and further promotes the development of depression (Penninx et al., 2004). Additionally, underlying diseases such as renal failure or inflammatory diseases could result in the development of both anemia and depression (Chen et al., 2010; Dorgalaleh et al., 2013; Fraenkel, 2017).

According to the theory of traditional Chinese medicine (TCM), depression could be ameliorated via modulating the blood system (Chen and You, 2005). It is believed that blood deficiency is the cause of recurrent melancholia (Cao, 2016). Therefore, hematinic and blood-nourishing medicines were usually used in classical anti-depressant formulas to enhance its therapeutic effect (Zhang, 2013). For example, *Ligusticum chuanxiong* Hort was used in Yueju, to regulate the blood system in depressed patients (Ren and Chen, 2017). Commonly, AS was used by traditional Chinese physicians to replenish the blood and promote circulation (Zhu et al., 2017). We speculated that the anti-depression effect of AS is related to the pharmacological activity of modulating the blood system.

Metabonomics, a crucial platform of systems biology, could explore the pathways associated with the pathological state and pharmacological action of drugs (Pferschy-Wenzig et al., 2017; Zhang et al., 2018). Located downstream of transcriptomics and proteomics, metabolomics reflects the terminal state of the metabolic network. Because it can evaluate the therapeutic effect by comprehensively detecting and quantifying the metabolite variations in biological systems, the method is especially suitable for the evaluation of the holistic and synergistic effects of TCM (Wu et al., 2018). Metabonomics has been used to explore the enriching blood mechanism of AS in previous reports (Li et al., 2014, 2015; Wang et al., 2016; Ji et al., 2018). In the current study, metabolomics was performed, based on ultra-performance liquid chromatography tandem mass spectrometry (UPLC-MS/MS) and nuclear magnetic resonance (NMR), to elucidate the anti-depression mechanisms of AS. The metabolites regulated by AS in depression were compared with that in anemia. The shared metabolites and pathways regulated in both the depression and anemia model, through the administration of AS, were further analyzed.

The aim of the current study is to elucidate the potential anti-depression mechanisms of AS that are related to the activity of modulating the blood system. Firstly, the model of CUMS was performed to assess the anti-depression effect of AS. Secondly, blood routine examinations and blood gas determinations were conducted to demonstrate that AS specifically reverses the disorder of the blood system induced by the CUMS procedure. Thirdly, a metabolomic approach based on UPLC-MS/MS combined with ^1^H NMR was employed to analyze the endogenous metabolites and metabolic pathways regulated by AS in depression. The disturbed metabolites and pathways were then regulated in an anemia model, through the administration of AS, summarized from previous literature and compared with that in depression in the present study. Finally, the expression of critical proteins on revealed signaling pathways, were further determined by western blotting.

## Materials and Methods

### Chemicals and Reagents

The radix of *Angelica sinensis* (*Oliv*.) Diels was purchased from the Shanxi Huayang Pharmaceutical Company and authenticated by Prof. Xue-Mei Qin (Shanxi University). Voucher specimens were deposited in the Modern Research Center for Traditional Chinese Medicine of Shanxi University. Chromatography grade methanol, acetonitrile and formic acid were obtained from Thermo Fisher Scientific Inc. (Waltham, MA, United States). Agar (Analytical grade). Deionized water was purified using a Milli-Q system (Billerica, MA, United States). i-STAT^®^ EG7+ was purchased from Abbott (Chicago, IL, United States). Venlafaxine hydrochloride was obtained from Kang Hong Pharmaceutical (Chengdu, China).

### Preparation of AS Extracts

The roots (4 kg) were extracted twice with 75% ethanol (1:8) under reflux, every time for 2 h, and the combined extracts were filtrated and concentrated in vacuo to a syrup. The syrup was then lyophilized into powders (48.1% yield).

### Animals and Drug Administration

A total of 60 healthy male Sprague-Dawley (SD) rats (weighing 180 ± 20 g) were obtained from the Beijing Vital Laboratory [Co. SCXK (Jing) 2016-0011]. All of the rats were adapted to the new experimental environment (12 h light dark cycle, 22 ± 2°C of room temperature) with free access to food and water for 1 week. The study was approved by the Experimental Animal Ethical Committee of Modern Research Center for Traditional Chinese Medicine, Shanxi University. All experimental procedures in the present study were performed in accordance with the NIH Guide for the Care and Use of Laboratory Animals (United States) and the Prevention of Cruelty to Animals Act (1986) of China.

**FIGURE 1:**
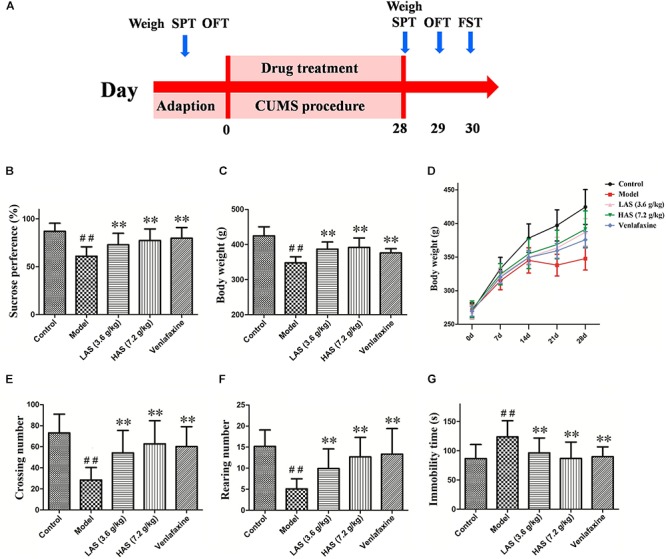
Effects of CUMS and drug treatment on behavior. **(A)** Study design. **(B)** Sucrose preference. **(C)** Body weight on day 28. **(D)** Body weight recorded weekly. **(E)** Crossing numbers in OFT. **(F)** Rearing numbers in OFT. **(G)** Immobility time in the FST. The data are presented as means ± SD (*n* = 12). ^##^*p* < 0.01 compared with control group; ^∗∗^*p* < 0.01 compared with the CUMS group.

After 1 week of acclimatization, the rats were randomly divided into five groups with 12 rats in each: the control (NS), model (MS), venlafaxine (VLF), high dose group of AS (HAS), and low dose group of AS (LAS) groups. The venlafaxine group of animals served as the positive control. For the next 28 days, the rats in the HAS and LAS group were given 15 and 7.5 g herb/kg AS according to the effective dose reported in previous research (Shen et al., 2016; Liu et al., 2017), the rats in the VLF group were intragastrically given 35 mg/kg venlafaxine. Animals were given the calculated amounts of fractions with the administration of a volume of 10 mL/kg (rat body weight), the rats in the control and model groups were given water. AS extract was dissolved in water with concentrations of 0.72 and 0.36 g/ml. Venlafaxine was dissolved in water with concentrations of 3.5 mg/ml. The experimental design of the study is depicted in Figure 1A.

### Chronic Unpredictable Mild Stress (CUMS) Procedure

The CUMS procedure was carried out as described previously (Tacchi et al., 2008; Gao et al., 2018), with minor modifications. The rats in the MS, VLF, HAS, and LAS groups were stimulated by the following stressors randomly between 9:00 and 11:00 a.m. every day and housed separately for 4 weeks. The stressors included: noises for 3 h (60 dB), swimming in the water at 4°Cfor 5 min, exposure to a hot room at 45°C for 10 min, deprivation for 24 h, food deprivation for 24 h, constraint for 2 h, tail clamp for 2 min, day–night reversal (12 h/12 h), unpredictable foot shocks for 2 min (36V, one shock/2 s, 10 s duration).

### Behavior Test

#### Open-Field Test (OFT)

The open-field test was, respectively, carried out at days 0, 7, 14, 21, and 28 to evaluate the general locomotor activity and depression-like behavior of rats (Zhou et al., 2011). The open field apparatus was a 100 cm × 100 cm × 40 cm black box containing 25 equal square regions that were defined by lines on the floor of the box. Every rat was placed into the center of the box and observed for 5 min. The crossing and rearing numbers were recorded.

#### Sucrose Preference Test (SPT)

The sucrose preference test was conducted at days 0 and 28 to quantify anhedonia, an important symptom of major depressive disorder (MDD). The test was performed as described previously (Zhou et al., 2011). All rats were trained before the test was carried out. Briefly, two bottles of sucrose solution (1%, w/v) were given to rats for 24 h, and then one of them was replaced with water for another 24 h. After a training session, the rats were deprived of water and food for 12 h, followed by free access to one bottle of a sucrose solution and another bottle of water for another 12 h. The weight of the consumed water and sucrose solution was measured and recorded. The sucrose preference rate was calculated as follows: consumed sucrose solution/(consumed sucrose + solution consumed water).

#### Forced Swim Test

The forced swim test was carried out as previously described (Zhang et al., 2017). The FST apparatus was a cylindrical container (50 cm in height and 20 cm in diameter) filled with water at 25 ± 2°C to a depth of 30 cm. Every rat was exposed to a pre-test for 15 min on the 1st day, and the FST was carried out on the next day. All rats were compelled to swim for 6 min. The immobility time was recorded during the last 4 min when the rats floated with their heads above the water and without struggling.

### Blood Collection, Blood Routine, and Blood Gas Determination

Twenty-four hours after the last intragastric administration, the blood of all rats was collected into a vacuum blood collection tube through an abdominal aortic method. 1.5 mL whole blood was collected into anticoagulant tubes containing ethylenediaminetetraacetic acid (EDTA) for peripheral blood routine examination by automated hematology analyzers. 1 mL whole blood was collected into heparinized anticoagulant tubes for blood gas determination. The remaining blood samples were collected into a vacuum blood collection tube without an anticoagulant, and immediately centrifuged at 3,000 rpm for 10 min. The serum was then collected into EP tubes and frozen at -80°C until LC-MS analysis.

### Metabolomics Study

#### Sample Preparation for Metabolomics Study

Serum samples were thawed at room temperature. For LC–MS metabolomics analysis, 100 μL serum samples were extracted with a mix of 225 μL methanol and 75 μL acetonitrile to precipitate protein, and the mixture was then vortexed for 2 min and centrifuged at 13,000 rpm for 15 min at 4°C. A quality control (QC) sample was prepared from 10 μL of each test sample. For NMR metabolomics analysis, 350 μL of D_2_O was added to 450 μL of the serum sample, and the mixture was then vortexed for 30 s, followed by centrifugation at 13,000 rpm for 20 min at 4°C. 550 μL of supernatant from the serum sample was transferred into a 5 mm NMR tube for NMR analysis.

#### LC–MS and NMR Method

A Dionex UltiMate 3000 UHPLC system combined with a Q Exactive Orbitrap-MS spectrometer and a Xcalibur workstation (Thermo Fisher Scientific Inc., Waltham, MA, United States) was used to acquire LC–MS data. An Acquity UPLC HSS T3 column was used for chromatographic separation. The mobile phase system included water/0.1% formic acid (solvent A) and acetonitrile/0.1% formic acid (solvent B) under a gradient elution as follows: 0 ∼ 2 min, 2% B; 2 ∼ 3 min, 2% B to 35% B; 3 ∼ 15 min, 35% B to 70% B; 15 ∼ 18 min, 70% B; 18 ∼ 29 min, 70% B to 98% B; 29 ∼ 31 min, 98% B; 31 ∼ 33 min, 98% B to 2% B; 33 ∼ 35 min, 2% B. The flow rate was set at 0.2 ml/min. The injection volume was 5 μL. All samples were analyzed under positive and negative ionization modes via a heated electrospray ionization (HESI) source. The detailed parameters were as follows: spray voltage of 3.5 kV for the positive mode and 2.5 kV for the negative mode, the capillary temperature of 320°C, the sheath of 35 arbitrary units, auxiliary gas flow rates of 10 arbitrary units. The range of mass scanning was set from 100 to 1500 (m/z).

The ^1^H NMR spectra were recorded at 298 K on a Bruker 600 MHz AVANCE III spectrometer (Bruker Biospin, Rheinstetten, Germany). Each ^1^H NMR spectrum included 64 scans acquired over 5 min. The detailed parameters were as follows: relaxation delay of 1.0 s, spectral size of 65536 points, and spectral width of 12019.2 Hz.

#### Data Analysis and the Screening of Potential Biomarkers

The acquired raw data from the LC–MS were introduced to the Compound Discoverer 2.0 (Thermo Fisher, United States) for peak alignment and detection. The primary parameters were: mass range, 100–1,500 Da; mass tolerance, 5 ppm; S/N threshold, 3; assignment threshold, 70. The peak area was normalized in Excel 2007. The obtained NMR spectra were introduced to MestReNova (version 8.0.1, Mestrelab Research, Santiago de Compostela, Spain) for phasing and correcting the baseline manually, and for referencing to the chemical shift of creatinine (3.04 ppm). Regions at δ 0.0–9.00 ppm were segmented at δ 0.01 intervals. Regions containing resonance from residual water (δ 4.50–5.00 ppm) were cut. The integral areas were then normalized to the total sum of spectra, to reduce the significant concentration differences.

The acquired data was imported into SIMCA-P V13.0 (Umetrics, Sweden) for multivariate statistical analysis. The different biological metabolites were selected based on VIP-value of S-plot (>1) and *T*-test (*p* < 0.05). The selected metabolites of the LC–MS analysis were identified according to the online databases: Metlin^1^, HMDB^2^, Massbank^3^, Pubchem^4^, Lipid Maps^5^ and KEGG^6^. The metabolites obtained from NMR analysis were identified based on moieties and chemical shifts. Pathway analysis was conducted with MetaboAnalyst^7^.

### Western Blot Analysis

The total proteins of the liver were extracted, and the concentrations were measured by a BCA procedure. Samples containing 50 μg proteins were separated by SDS-PAGE electrophoresis and transferred to PVDF membranes. The membranes were blocked with 5% bovine albumin (BSA) in Tris buffer saline-Tween 20 (TBST) for 2 h at 37°C, and then incubated overnight at 4°C with the respective first antibodies diluted in 1.5: 1000. After washing with TBST, the membranes were incubated with fluorescent secondary antibodies (1: 15000) for 2 h at 37°C. After rewashing with TBST, the membranes were scanned and visualized by a fluorescent scanner (Odyssey CLX, Gene Company Limited, United States).

### Statistical Analysis

Statistical analysis was performed using SPSS 16.0 software, and all data were expressed as mean ± standard deviation (SD). Statistical differences between two groups were compared by *T*-test and the significant differences between more groups were compared by one-way ANOVA. A value of *p* < 0.05 was regarded as a significant difference.

## Results

### The Anti-depression Effects of Angelicae Sinensis Radix on Behavior

On day 0, no significant difference in sucrose preference, body weight or SPT were observed among the five groups (data not shown). After a 28-day CUMS treatment, the model group showed a significant decrease in sucrose preference compared with the control group (Figure 1B). The treatment group of AS and VLF resulted in a significant increase in sucrose preference compared with the CUMS group, indicating a reduction of anhedonia. The result of body weight is shown in Figure 1C,D. At the beginning of the experiment, no differences in body weight was shown among the five groups. On day 7, there were significant differences between the control and CUMS group. On days 21 and 28, the AS and VLF groups showed significantly higher body weight compared with the CUMS group. Similar results were observed in the OFT (Figure 1E,F) and FST (Figure 1G). After 28 days of the CUMS procedure, the crossing and rearing numbers in OFT were markedly lower, and the immobility time in FST was markedly longer in the CUMS group than the control group. After the treatment with AS and VLF for 28 days, these changes induced by CUMS were significantly attenuated.

### Angelicae Sinensis Radix Reversed the Disorder of Peripheral Blood Routine Induced by CUMS

**FIGURE 2:**
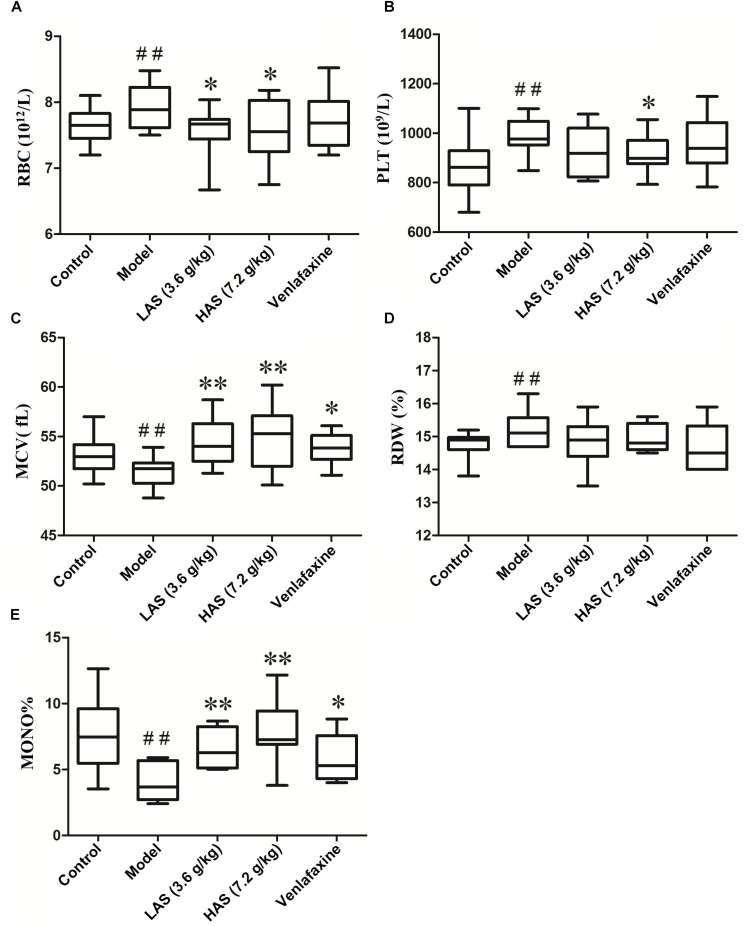
Effects of CUMS and drug treatment on peripheral blood routine. **(A)** Erythrocyte count (RBC). **(B)** Platelet count (PLT). **(C)** Erythrocyte mean corpuscular volume (MCV). **(D)** Red blood cell distribution width (RDW) **(E)** Mono%. The data are presented as means ± SD (*n* = 12). ^##^*p* < 0.01 compared with control group; ^∗^*p* < 0.05, ^∗∗^*p* < 0.01 compared with the CUMS group.

The results of peripheral blood routine analysis is presented in Figure 2A–E. Compared with the control group, the level of red blood cells (RBC), platelet count (PLT), and red blood cell distribution width (RDW) in the CUMS group was significantly increased, while the level of monocyte proportion (MO%) and mean corpuscular volume (MCV) in the CUMS group was significantly decreased. After oral administration of AS, the reduced MO% and MCV were markedly increased, while elevated RBC and PLT were markedly decreased compared with the CUMS group. The administration of VLF could only reverse the abnormal peripheral blood indicators induced by on the CUMS procedure on MO% and MCV.

### Angelicae Sinensis Radix Reversed the Disorder of Blood Gas Induced by CUMS

**FIGURE 3:**
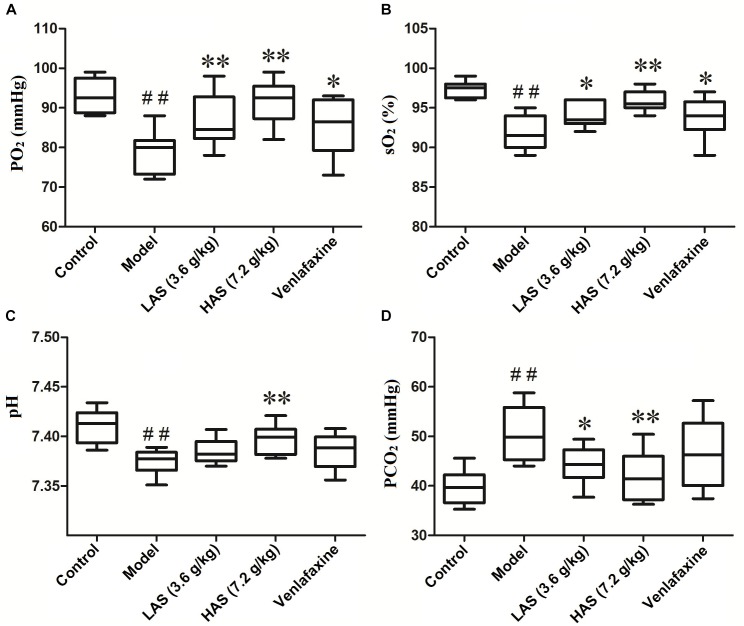
Effects of CUMS and drug treatment on blood gas. **(A)** Partial pressure of oxygen (PO_2_). **(B)** Oxygen saturation (sO_2_). **(C)** Hydrogen ion concentration (pH). **(D)** Partial pressure of carbon dioxide (PCO_2_). The data are presented as means ± SD (*n* = 8). ^##^*p* < 0.01 compared with control group; ^∗^*p* < 0.05, ^∗∗^*p* < 0.01 compared with the CUMS group.

The blood gas determination was performed on a blood gas analyzer (i-STAT 300, Abbott, United States). As presented in Figure 3A,B, the partial pressure of oxygen (PO_2_) and oxygen saturation (sO_2_) in the CUMS group was significantly decreased compared with the control group, suggesting that depression is accompanied by hypoxia. On the contrary, the level of PO_2_ and sO_2_ was significantly increased after the treatment with AS and VLF. Figure 3C,D shows that the CUMS treatment of rats for 28 days resulted in a significant increase in partial pressure of carbon dioxide (PCO_2_) and a significant decrease in hydrogen ion concentration (pH), indicating an occurrence of electrolyte disturbance. However, the phenomenon was reversed after the administration of AS.

### Angelicae Sinensis Radix Modulated the Blood System in CUMS-Induced Rats

#### Multivariate Data Analysis

**FIGURE 4:**
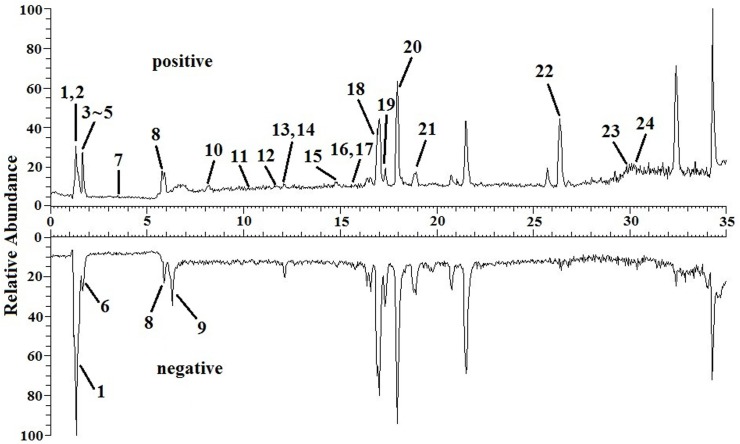
Total positive ions and negative ions UPLC-MS/MS chromatograms of serum sample with the differential metabolites labeled. Numbers represent differential metabolites identified between the CUMS group and the control group.

**FIGURE 5:**
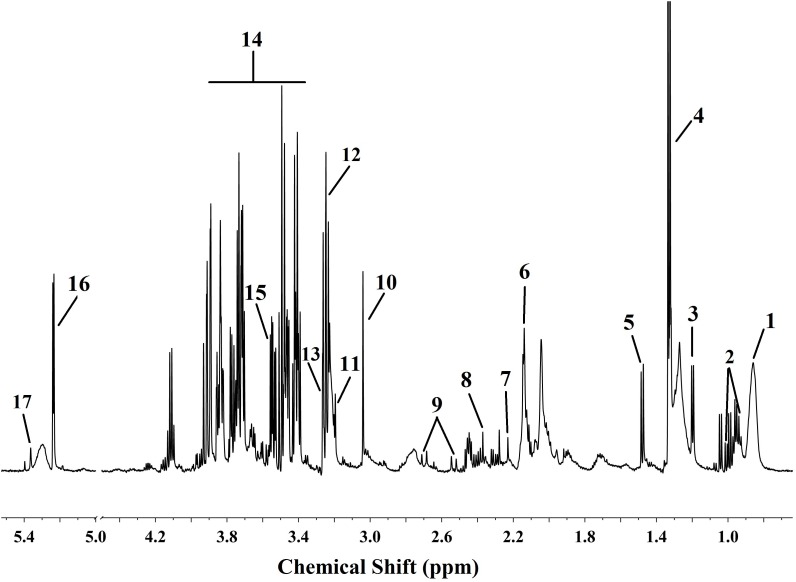
Typical ^1^H NMR (600 MHz) spectroscopy of serum sample with the differential metabolites labeled. Numbers represent differential metabolites identified between the CUMS group and the control group.

**FIGURE 6:**
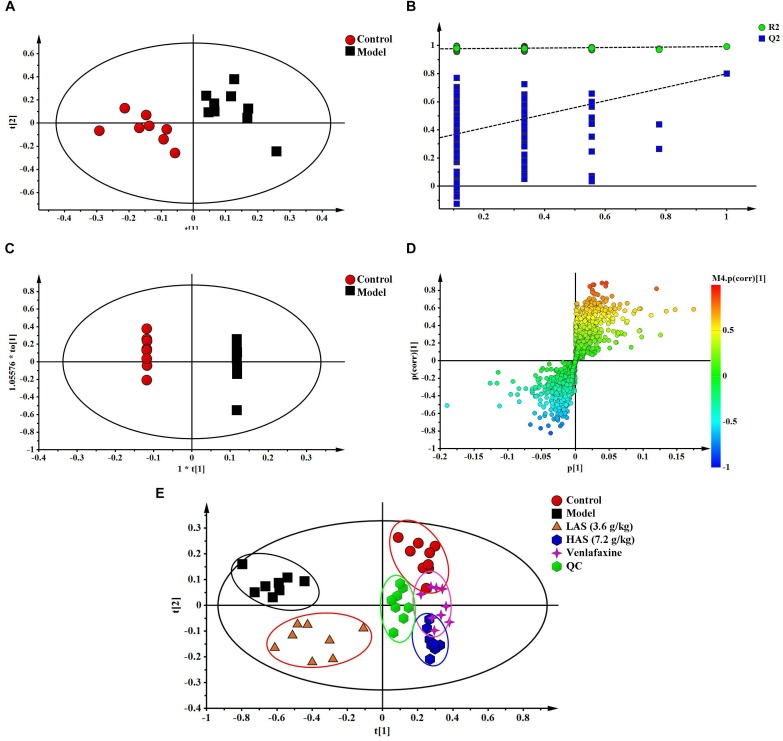
Multivariate data analysis from UPLC-MS/MS. **(A)** PLS-DA score plots, **(B)** PLS-DA model validation diagram, **(C)** OPLS-DA score plots, **(D)** S-plot of OPLS-DA, **(E)** PLS-DA score plots of serum samples collected from different groups.

**FIGURE 7:**
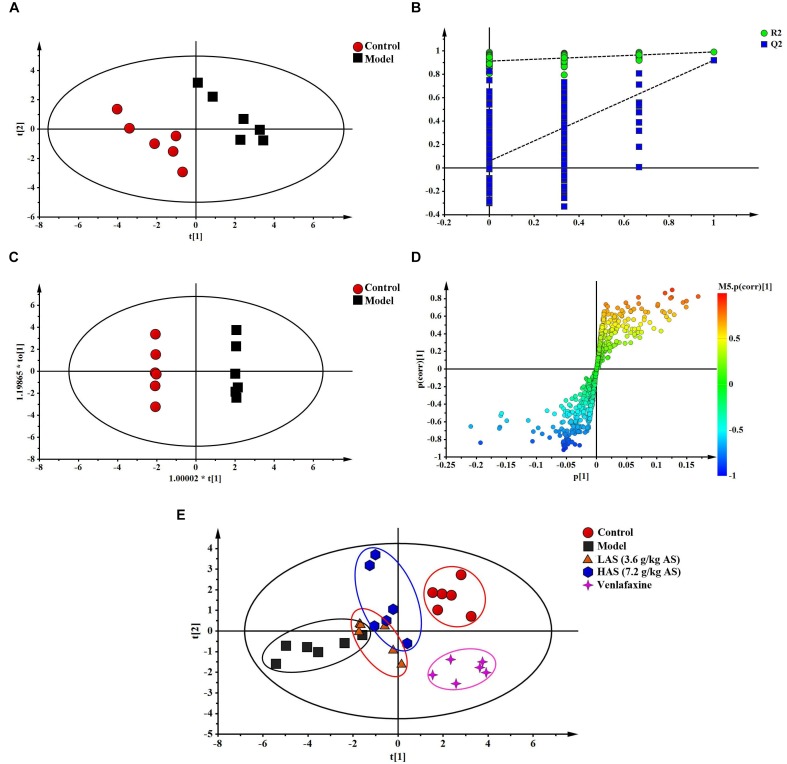
Multivariate data analysis from NMR. **(A)** PLS-DA score plots, **(B)** PLS-DA model validation diagram, **(C)** OPLS-DA score plots, **(D)** S-plot of OPLS-DA, **(E)** PLS-DA score plots of serum samples collected from different groups.

The TIC chromatograms of serum samples from UPLC-MS/MS in both positive and negative modes are shown in Figure 4, and a representative ^1^H NMR spectra of serum samples is shown in Figure 5. The variables were, respectively, obtained from Compound Discoverer 2.0 and MestReNova software. These variables were then imported into SIMCA-P V13.0 for further multivariate statistical analysis. The partial least squares-discriminant analysis (PLS-DA) score plots from LC-MS and NMR both demonstrated that the rats in the CUMS group was obviously separated from the rats in the control group (Figure 6A, 7A). The PLS-DA model was validated using the response of the permutation test (Figure 6B, 7B). R^2^X of the PLS-DA model in LC-MS and NMR was 0.532 and 0.675, respectively; R^2^Y was 0.984 and 0.991; and *Q*^2^ was 0.699 and 0.92, suggesting that the model was excellent for prediction. The orthogonal partial least squares discriminant analysis (OPLS-DA) model was then applied to discriminate the differential metabolites contributing to the separation of the CUMS group and the control group. As shown in Figure 6C, 7C, significant separations between the CUMS and the control group were observed in the OPLS-DA score plots. The S-plots of OPLS-DA revealed the variation of metabolites as shown in Figure 6D, 7D. PLS-DA was future performed to investigate the regulatory effect of AS and VLF. Figure 6E, 7E show that all experimental groups were obviously separated in both the LC-MS and NMR metabolic profile. Among them, the metabolic profiles in the AS and VLF group were closer to the control group than the CUMS group, suggesting that the metabolic disturbances induced by CUMS were reversed after drug treatment.

#### Identification of Endogenous Metabolites

**FIGURE 8:**
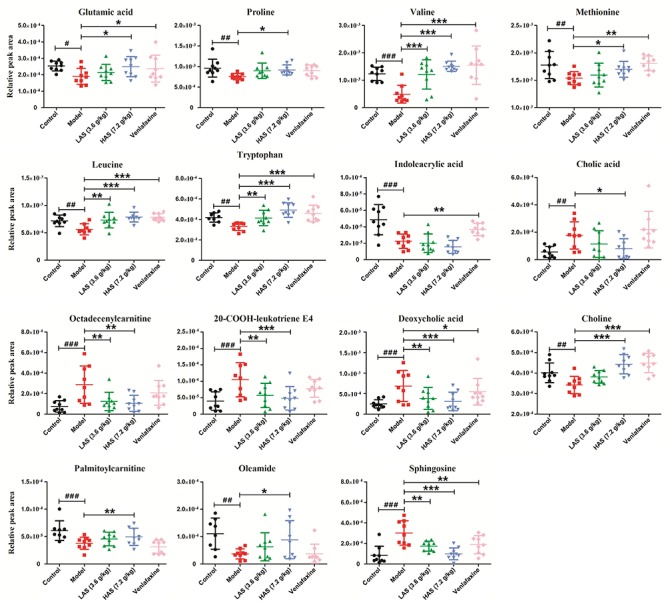
Comparison of relative peak areas of the differential metabolites in UPLC-MS/MS associated with drug treatment. The data are presented as means ± SD (*n* = 9). ^#^*p* < 0.05, ^##^*p* < 0.01, ^###^*p* < 0.001 compared with control group; ^∗^*p* < 0.05, ^∗∗^*p* < 0.01, ^∗∗∗^*p* < 0.001 compared with the CUMS group.

**FIGURE 9:**
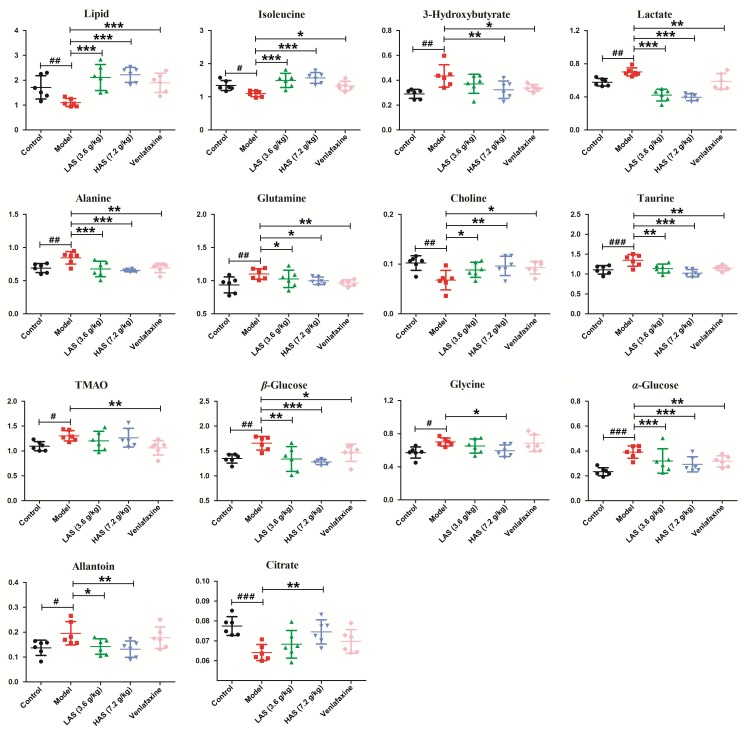
Comparison of the relative intensity of differential metabolites in NMR associated with drug treatment. The data are presented as means ± SD (*n* = 6). ^#^*p* < 0.05, ^##^*p* < 0.01, ^###^*p* < 0.001 compared with control group; ^∗^*p* < 0.05, ^∗∗^*p* < 0.01, ^∗∗∗^*p* < 0.001 compared with the CUMS group.

Significantly differential metabolites were screened according to the VIP values of S-plots (>1.0) and *T*-tests (*p* < 0.05). The metabolites obtained from LC-MS analysis were identified according to MS/MS fragments, retention behavior and online databases. As a consequence, a total of 24 endogenous biomarkers in serum were screened (Table 1). The metabolites obtained from NMR analysis were identified based on moieties and chemical shifts, and 17 endogenous biomarkers were screened (Table 2). The level of 3-Hydroxybutyrate, lactate, alanine, glutamine, taurine, TMAO, β-Glucose, glycine, α-Glucose, allantoin, pipecolic acid, 3-Indoxyl sulfate, cholic acid, phytosphingosine, octadecenylcarnitine, 20-COOH-leukotriene E4, sphingosine, LysoPC (20:5), deoxycholic acid, LysoPC (16:0), and 20-Oxo-leukotriene E4 was significantly increased in the CUMS group as compared to the control group. The level of lipid, isoleucine, acetone, pyruvate, citrate, creatine, choline, glutamic acid, proline, valine, methionine, propionylcarnitine, leucine, tryptophan, indoleacrylic acid, palmitoylcarnitine, oleamide, and stearamide was significantly decreased in the CUMS group as compared to the control group. The variation of endogenous biomarkers from LC-MS is illustrated in Figure 8, and that from NMR is illustrated in Figure 9. A total of 26 metabolites including lipid, isoleucine, 3-Hydroxybutyrate, lactate, alanine, citrate, glutamine, choline, taurine, β-Glucose, glycine, α-Glucose, allantoin, glutamic acid, proline, valine, methionine, leucine, tryptophan, cholic acid, octadecenylcarnitine, 20-COOH-leukotriene E4, sphingosine, deoxycholic acid, oleamide, and palmitoylcarnitine was intervened by AS treatment. VLF treatment also exerted an effect on these metabolic alterations to a certain extent, while the effect was weaker than AS.

**Table 1:** Differential metabolites associated with depression were detected by UPLC-MS/MS.

No.	Metabolites	*T*_R_ (min)	m/z	Formula	VIP	*P*	Fold change	Trend	HMDB ID	Scan mode
(1)	Glutamic acid^a^	1.31	148.0604	C_5_H_9_NO_4_	1.41	^∗∗^	0.75	↓	00148	+
(2)	Proline	1.42	116.0706	C_5_H_9_NO_2_	2.60	^∗^	0.78	↓	00162	+
(3)	Valine^a^	1.50	118.0863	C_5_H_11_NO_2_	4.17	^∗∗∗^	0.48	↓	00883	+
(4)	Methionine	1.65	150.0583	C_5_ H_11_ N O_2_ S	2.56	^∗^	0.86	↓	00696	+
(5)	Propionylcarnitine	1.66	218.1387	C_10_H_19_NO_4_	1.83	^∗^	0.71	↓	00824	+
(6)	Pipecolic acid	1.67	128.0717	C_6_H_11_NO_2_	2.12	^∗∗^	2.02	↑	00070	-
(7)	Leucine^a^	3.49	132.1019	C_6_H_13_NO_2_	1.52	^∗∗∗^	0.79	↓	00687	+
(8)	Tryptophan^a^	5.75	205.0972	C_11_H_12_N_2_O_2_	1.24	^∗∗∗^	0.79	↓	00929	+
(9)	3-Indoxyl sulfate	6.30	212.0023	C_8_H_7_NO_4_S	2.28	^∗∗^	1.96	↑	00682	-
(10)	Indoleacrylic acid	7.92	188.0706	C_11_H_9_NO_2_	1.16	^∗∗^	0.51	↓	00734	+
(11)	Cholic acid	10.19	409.2949	C_24_H_40_O_5_	2.29	^∗∗^	3.18	↑	00619	+
(12)	Phytosphingosine	11.67	318.3003	C_18_H_39_NO_3_	1.13	^∗^	1.51	↑	04610	+
(13)	Octadecenylcarnitine	12.06	426.3581	C_24_H_43_NO_5_	3.25	^∗∗^	3.81	↑	13338	+
(14)	20-COOH-leukotriene E4	12.07	470.2583	C_24_H_41_NO_4_P_2_	1.75	^∗∗^	2.63	↑	12634	+
(15)	Sphingosine	15.30	300.2897	C_18_ H_37_ N O_2_	1.70	^∗∗∗^	3.54	↑	00252	+
(16)	LysoPC (20:5)	15.44	542.3241	C_28_H_48_NO_7_P	1.39	^∗∗^	1.41	↑	10397	+
(17)	Deoxycholic acid	15.65	393.2999	C_24_H_40_O_4_	2.90	^∗∗^	2.74	↑	00626	+
(18)	LysoPC (16:0)	17.27	496.3403	C_24_H_50_NO_7_P	4.13	^∗^	1.10	↑	10382	+
(19)	20-Oxo-leukotriene E4	17.75	454.2928	C_21_H_44_NO_7_P	3.02	^∗∗∗^	1.25	↑	12642	+
(20)	Choline	17.92	104.1070	C_5_H_13_NO	1.73	^∗∗∗^	0.85	↓	00097	+
(21)	Palmitoylcarnitine	18.53	400.3421	C_23_H_45_NO_4_	1.28	^∗∗^	0.62	↓	00222	+
(22)	Oleamide	26.70	282.2791	C_18_H_35_NO	1.81	^∗∗^	0.33	↓	02117	+
(23)	Stearamide	29.02	284.2948	C_18_H_37_NO	2.81	^∗^	0.53	↓	34146	+
(24)	Palmitoyl sphingomyelin	30.98	703.5749	C_39_H_79_N_2_O_6_P	1.10	^∗^	1.43	↑		+


*“↓” or “↑” means the metabolite significantly decreased or increased in the CUMS group compared with control group. ^∗^p < 0.05, ^∗∗^p < 0.01, ^∗∗∗^p < 0.001 compared with control group. ^*a*^Validated with standard.*

**Table 2:** Differential metabolites associated with depression were detected by NMR.

No.	Metabolites	Chemical shift	VIP	*P*	Fold change	Trend	HMDB ID
(1)	Lipid	0.86(m), 1.28(m), 2.78(m), 5.30(m)	4.64	^∗^	0.64	↓	13244
(2)	Isoleucine	0.94(t), 1.01(d), 1.27(m)	3.31	^∗∗^	0.81	↓	00172
(3)	3-hydroxybutyrate	1.20(d), 2.41(d), 2.31(d)	2.03	^∗∗^	1.50	↑	00357
(4)	Lactate	1.33(d), 4.12(q)	3.10	^∗∗^	1.21	↑	00190
(5)	Alanine	1.48(d), 3.77(q)	1.48	^∗∗^	1.22	↑	00161
(6)	Glutamine	2.14(m), 2.46(m)	3.21	^∗^	1.18	↑	00641
(7)	Acetone	2.23(s)	1.67	^∗^	0.72	↓	01659
(8)	Pyruvate	2.37(s)	2.28	^∗∗^	0.69	↓	00243
(9)	Citrate	2.54(d), 2.70(d)	1.14	^∗∗∗^	0.82	↓	00094
(10)	Creatine	3.04(s), 3.94(s)	2.84	^∗∗^	0.74	↓	00064
(11)	Choline	3.20(s)	1.56	^∗∗^	0.66	↓	00097
(12)	Taurine	3.26(t), 3.41(t)	3.55	^∗^	1.26	↑	00251
(13)	TMAO	3.27(s)	3.27	^∗∗^	1.19	↑	00925
(14)	β-Glucose	3.49(t), 3.41(dd), 3.73(dd), 3.90(dd)	4.92	^∗∗^	1.23	↑	00122
(15)	Glycine	3.56(s)	2.30	^∗∗^	1.22	↑	00123
(16)	α-Glucose	5.24(d), 3.54(d)	3.67	^∗∗∗^	1.67	↑	00122
(17)	Allantoin	5.38(s)	1.46	^∗^	1.43	↑	00462


*“↓” or “↑” means the metabolite significantly decreased or increased in the CUMS group compared with control group. ^∗^p < 0.05, ^∗∗^p < 0.01, ^∗∗∗^p < 0.001 compared with control group.*

#### Metabolic Pathway Analysis

**FIGURE 10:**
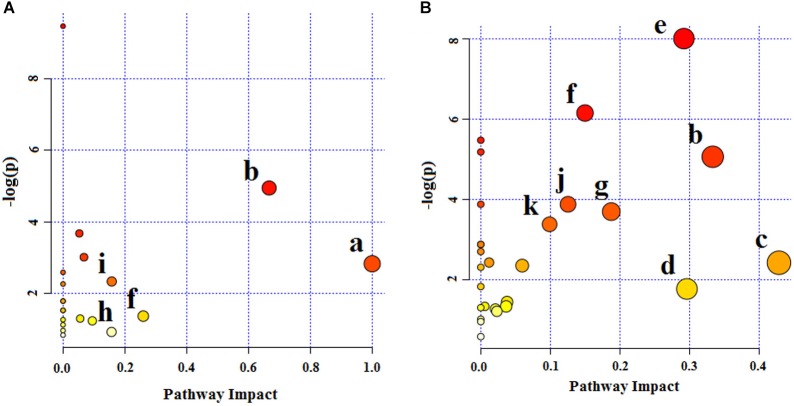
Summary of pathway analysis of serum samples collected from CUMS rats. **(A)** Pathway analysis from UPLC-MS/MS. **(B)** Pathway analysis from NMR. (a) D-Glutamine and D-glutamate metabolism, (b) valine, leucine and isoleucine biosynthesis, (c) taurine and hypotaurine metabolism, (d) glyoxylate and dicarboxylate metabolism, (e) glycine, serine and threonine metabolism, (f) alanine, aspartate and glutamate metabolism, (g) pyruvate metabolism, (h) tryptophan metabolism, (i) arginine and proline metabolism, (j) TCA cycle, (k) glycolysis or gluconeogenesis metabolism.

Twenty-four endogenous biomarkers identified from LC-MS and 16 from NMR were, respectively, imported into the MetaboAnalyst, to explore the potential metabolic pathways of depression. The metabolic pathways are summarized in Figure 10. According to the biomarkers identified from LC-MS, D-Glutamine and D-glutamate metabolism with an impact-value of 1.0, valine, leucine and isoleucine biosynthesis with an impact-value of 0.67, alanine, aspartate and glutamate metabolism with an impact-value of 0.26, tryptophan metabolism with an impact-value of 0.16, and arginine and proline metabolism with an impact-value of 0.16 were filtered out as the most significant metabolic pathways with an impact-value over 0.10. Based on the biomarkers identified from NMR, the main changed metabolic pathways of depression were taurine and hypotaurine metabolism (impact-value 0.43); valine, leucine and isoleucine biosynthesis (impact-value 0.33); glyoxylate and dicarboxylate metabolism (impact-value 0.30); glycine, serine and threonine metabolism (impact-value 0.29); pyruvate metabolism (impact-value 0.19); alanine, aspartate and glutamate metabolism (impact-value 0.15); citrate cycle (impact-value 0.13); and glycolysis or gluconeogenesis (impact-value 0.10).

### The Relation of Disturbed Metabolites Related by Angelicae Sinensis Radix Between Depression and Anemia

**FIGURE 11:**
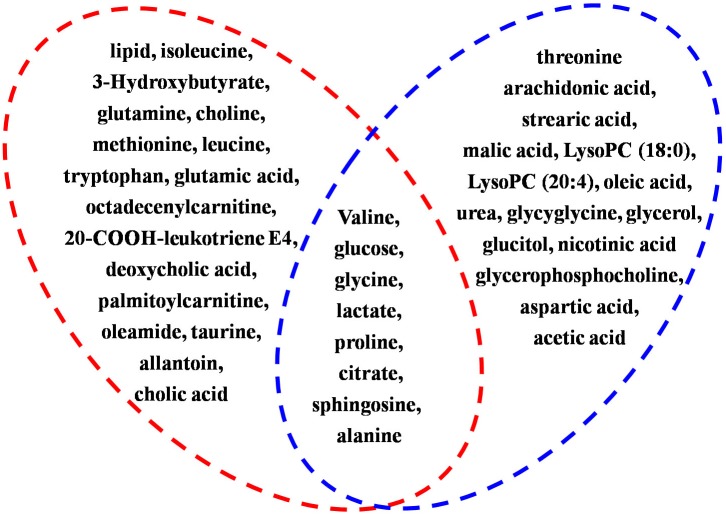
Summary of shared metabolites regulated by AS both in depression and anemia. Metabolites in red circle represents the disturbed metabolites regulated by AS in depression, metabolites in blue circle represents the disturbed metabolites regulated by AS in anemia. Eight biomarkers included valine, glucose, glycine, lactate, proline, citrate, sphingosine, and alanine are the shared metabolites.

**FIGURE 12:**
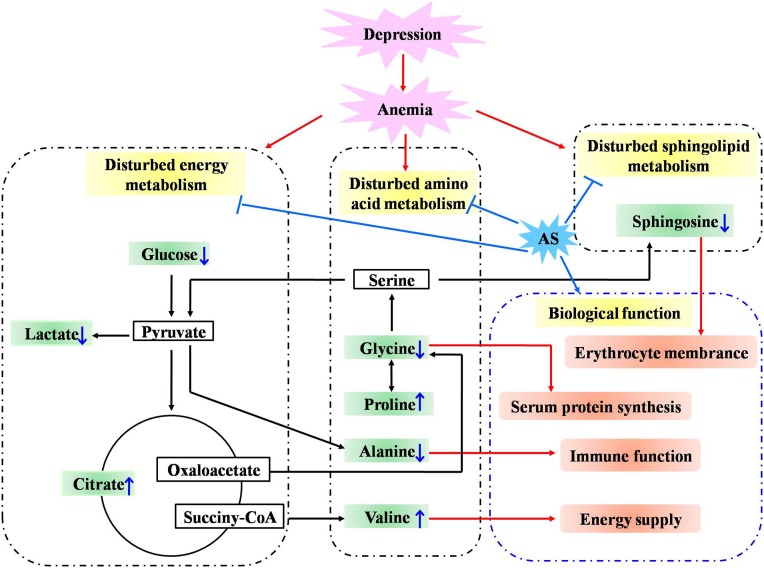
Potential anti-depression mechanisms of AS related to the activity of modulating the blood system. “↓” or “↑” means the metabolite significantly decreased or increased in AS group compared with the CUMS group.

To investigate the linkage between the effect of anti-depression and modulating the blood system of AS, the disturbed metabolites regulated by AS in anemia were mined according to previous literature. Briefly, two keywords including AS and metabonomics were inputted into PubMed^8^ to search the literature, the obtained literature associated with anemia was further filtrated out. As a result, five studies in the literature, which reported that disturbed metabolites were regulated in anemia through the administration of AS, were screened, and these metabolites were further compared with those regulated in depression by AS in the current study. As presented in Figure 11, a total of eight biomarkers including valine, glucose, glycine, lactate, proline, citrate, sphingosine, and alanine were regulated by AS both in depression and anemia. These metabolites mainly participate in the metabolic pathways including the TCA cycle, amino acid, carbohydrate and sphingolipid metabolism. The mechanism may be associated with the promotion of the body’s energy metabolism, the stabilization of the cell membrane, the promotion of serum protein synthesis, and the enhancement of immunity (Figure 12).

### Effects of Angelicae Sinensis Radix on the Expression of LDHA and PDK-1

**FIGURE 13:**
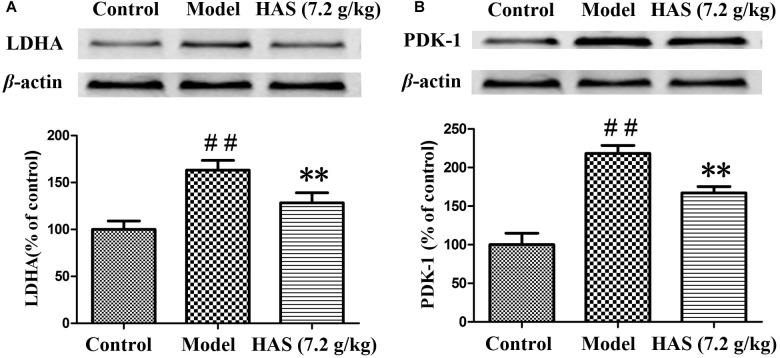
Effects of Angelicae Sinensis Radix on the expression level of LDHA and PDK-1. **(A)** LDHA proteins. **(B)** PDK-1 proteins. The data are presented as means ± SD. ^##^*p* < 0.01 compared with control group; ^∗∗^*p* < 0.01 compared with the CUMS group.

The expression of critical proteins on energy metabolism was further determined to validate the metabolic results. PDK-1 could inhibit the activity of pyruvate dehydrogenase complex (PDC) and block the production of acetyl coenzyme A, which acts as an intermediate metabolite of energy metabolism. LDHA catalyzes the interconversion of pyruvate and NADH to lactate and NAD^+^. The results are presented in Figure 13A,B. The level of PDK-1 and LDHA was significantly elevated in the liver of rats in the CUMS group compared with that in the control group, suggesting that energy metabolism was disrupted after the CUMS procedure. However, the elevated level of PDK-1 and LDHA was obviously decreased after the treatment with AS. The results proved that AS could relieve depression syndrome by regulating energy metabolism.

## Discussion

Angelicae Sinensis Radix, a famous TCM herb wildly used in Asia for thousands of years, has in recent years been confirmed to have an anti-depression. Our present study demonstrates that AS significantly improves the depressive symptom induced by CUMS as described in previous reports (Shen et al., 2016). AS could significantly increase the decreased body weight, sucrose preference and locomotor activity induced by the CUMS procedure, and reduce the increased immobility time in FST induced by the CUMS procedure. AS (low dose and high dose) could increase a more reduced body weight than VLF. It is speculated that AS extract is rich in polysaccharides, which could turn to fat *in vivo*.

Accumulating evidence has suggested that depressive symptoms are associated with anemia. It has been proposed that depression could be ameliorated via modulating the blood system. AS is applied for invigorating blood circulation and enriching blood traditionally. Therefore, we speculated that the anti-depression effect of AS may be related to the pharmacological activity of modulating the blood system. In the current study, our results demonstrated that the blood circulation system was exactly disordered by the CUMS procedure, which could be significantly reversed by the treatment of AS. AS showed a higher activity than VLF on the effect of regulating the blood system, which is in accordance with the “multi-component and multi-target pattern” of TCM.

Red blood cell distribution width is a parameter representing the coefficient of variation of the red blood cell volume distribution and can be considered as a more sensitive indicator to establish the origin of microcytic hypochromic anemia (Aulakh et al., 2009). RDW and MCV can be applied for the morphological classification of anemia (Buttarello, 2016). In the current study, the level of MCV was decreased and the level of RDW was increased in the model group, suggesting that iron deficiency anemia occurred in the CUMS procedure (Urrechaga et al., 2015). These results agrees with the literature, i.e., RDW level was elevated and MCV level was reduced in depressive patients as evidenced in previous reports (Fatih et al., 2016; Cai et al., 2017). In this study, we discovered that anemia accompanied depression, however, the level of RBC was conversely increased in the model group and no significant difference was observed in hemoglobin concentration (Hb) between the CUMS and control group (data not shown). This may be because in the early period of iron deficiency anemia, the absence of iron results in a reduction of heme and hemoglobin, which induces a compensatory elevation of RBC (Joharapurkar et al., 2018). On the other hand, the elevation of RBC in the CUMS group could be partly ascribed to stress polycythemia (Gaisböck syndrome), a pathological state with abnormally increasing erythrocyte in the blood which is often seen in distorted personality (nervosity, cyclothymia, or depression) or active individuals under emotional stress (Murakami et al., 1999). Additionally, thrombocythemia is often seen in erythrocytosis. The elevated PLT in the CUMS group could therefore be associated with the elevation of RBC. In the current study, we found that these disordered peripheral blood indicators were regulated to a normal level after the administration of AS.

**FIGURE 14:**
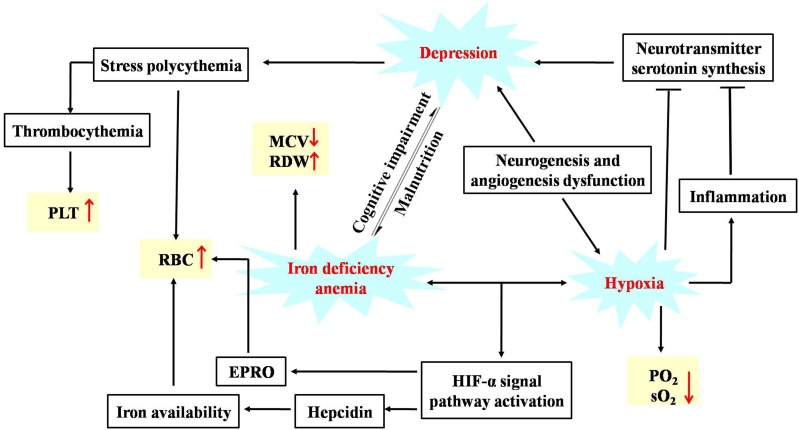
Graphic representation of the hypotheses for the association of depression, anemia and hypoxia.

An increasing body of research has indicated that vascular dysfunction plays a crucial role in the development of MDD. Angiogenesis and neurogenesis is interdependent in the neurogenic/neurotrophic theory of depression. The trophic factors VEGF may serve as a common thread that connects angiogenesis and neurogenesis (Fournier and Duman, 2012). Hypoxia is a common finding in depression, which is ascribed to capillary dysfunction (Østergaard et al., 2018). Inflammation is triggered to help tissues adapt to hypoxia. The vicious spiral of capillary dysfunction, hypoxia and inflammation affects the synthesis of the neurotransmitter serotonin, which finally accelerates the development of depression. Graphic representation of the hypotheses for the association of depression, anemia and hypoxia was shown in Figure 14. Our current study suggests that obvious symptoms of hypoxia were observed after the CUMS procedure which could be improved by the treatment of AS, due to the pharmacological action of AS of promoting capillary generation.

Hyperactivity of the hypothalamic-pituitary-axis has commonly been considered as the pathogenesis of major depression. Evidence has suggested that the abnormality of energy metabolism in depression may be associated with excess glucocorticoids (GC). On the one hand, high levels of GC exposure result in the deterioration of mitochondrial function by interfering with the proton gradients across their mitochondria’s inner membranes (Du et al., 2009; Hunter et al., 2016). On the other hand, high levels of GC affect the body’s energy expenditures. The body’s energy balance shifts from anabolism toward catabolism, which finally results in lipolysis in adipose tissue and protein degradation in muscle and bones, to mobilize available energy resources (Østergaard et al., 2018). Our metabonomics results demonstrated that metabolites such as pyruvate, citrate, lactate, and glucose, involved in energy metabolism, were disturbed in the CUMS group, indicating an imbalance of energy metabolism, which may be due to hypoxia and the excess of GC. The level of citrate, lactate and glucose was significantly reversed by the administration of AS, suggesting that AS could relieve depression syndrome by improving energy metabolism. Compared with the AS group, the oxygen content level (PO_2_ and sO_2_) upregulated by VLF was lower. The lower oxygen content in the VLF group resulted in a disorder of the TCA cycle, followed by a decrease of citrate. Therefore, the level of citrate was higher in the AS group than that in the VLF group. The decreased oxygen content in the VLF group resulted in a promotion of anaerobic metabolism, which was followed by a lactate elevation. As a result, the abnormally decreased pH value could not be significantly reversed by the administration of VLF.

Sphingolipids play a crucial role in the composition of the cell membrane and participate in the many signaling processes, such as cell growth, differentiation, and programmed cell death (Testai et al., 2014). Sphinganine, sphinganine 1-phosphate, ceramide, and phytosphingosine are disturbed during the process of anemia, which may be associated with the injured erythrocyte membrane and disordered peripheral blood indicators (Li et al., 2014; Pang et al., 2018). AS could significantly down-regulate the increased sphingolipids and phytosphingosine induced by anemia, suggesting that AS could relieve anemia syndrome by regulating the sphingolipid metabolism. In the current study, we demonstrated that sphinganine and phytosphingosine were significantly increased in the CUMS group, while sphinganine was significantly decreased after the administration of AS. Sphingolipid metabolism acts as a common thread that connects depression and anemia. The syndrome of anemia, accompanied by depression could be relieved by AS through sphingolipid metabolism, which finally results in the amelioration of depression. A down-regulation tendency in sphingosine, which participates in the composition of the cell membrane (Testai et al., 2014), was also observed in the VLF group. However, the effect was weaker than that in the AS group. Thus, the modulation effect of VLF on RBC composed of sphingosine, was also weaker than that of AS.

Several amino acids have been regarded as important biomarkers related to anemia. In the case of blood deficiency, valine regulates blood sugar levels and supply extra energy to muscles (Allen et al., 2015). Thus, valine is significantly decreased in the model of anemia. Glycine has been reported to regulate serum iron and promote the synthesis of serum protein. The elevation of the glycine level in the case of anemia could be associated with the protection of ischemic cell and the improvement of immune function (Li et al., 2013). Alanine plays a crucial role in immune function and the regeneration of lymphocytes (Newsholme et al., 2007). The reduction of alanine in the case of anemia could be related to damage of the immune function. In the current study, we found that nine amino acids were disturbed in the procedure of CUMS, indicating a disturbance of amino acid metabolism. In accordance with the disturbed amino acids regulated by AS in anemia, four amino acids including valine, glycine, alanine, and proline were also regulated by the administration of AS in depression. These finding suggested that the syndrome of anemia accompanied by depression could be relieved by AS, through an amino acid metabolism, which finally results in the amelioration of depression.

## Conclusion

Angelicae Sinensis Radix could significantly improve the CUMS-induced depressive symptoms, anemia symptoms, and hypoxia symptoms. The analysis of metabonomics suggested that the anti-depression effect of AS was related to the function of modulating the blood system, which is mainly associated with the metabolic pathways including energy, amino acid, and sphingolipid metabolism. The mechanism may be associated with the promotion of the body’s energy metabolism, the stabilization of the cell membrane, the promotion of serum protein synthesis, and the enhancement of immunity.

## Author Contributions

YZ, XQ, and GD conceived and designed the experiments. WG, CC, SZ, and QY performed the experiments. XL assisted in the testing and analysis of data. WG and YZ drafted the manuscript.

## Conflict of Interest Statement

The authors declare that the research was conducted in the absence of any commercial or financial relationships that could be construed as a potential conflict of interest.

## Footnotes

1 http://metlin.scripps.edu

2 http://www.hmdb.ca

3 http://www.massbank.jp

4 https://pubchem.ncbi.nlm.nih.gov/

5 http://www.lipidmaps.org

6 http://www.kegg.jp

7 http://www.metaboanalyst.ca/

8 https://www.ncbi.nlm.nih.gov/pubmed

## References

[B1] Allen P. J. Wise D. Greenway T. (2015). Using 1-D 1H and 2-D 1H J-resolved NMR metabolomics to understand the effects of anemia in channel catfish (*Ictalurus punctatus*). *Metabolomics* 11 1131–1143.

[B2] Aulakh R. Sohi I. Singh T. Kakkar N. (2009). Red cell distribution width (RDW) in the diagnosis of iron deficiency with microcytic hypochromic anemia. *Indian. J. Pediatr.* 76 265–268. 10.1007/s12098-009-0014-4 19205647

[B3] Buttarello M. (2016). Laboratory diagnosis of anemia: are the old and new red cell parameters useful in classification and treatment, how? *Int. J. Lab. Hematol.* 38 123–132. 10.1111/ijlh.12500 27195903

[B4] Cai L. Xu L. Wei L. Chen W. (2017). Relationship of mean platelet volume to MDD: a retrospective study. *Shanghai Arch. Psychiatry* 29 21–29. 10.11919/j.issn.1002-0829.216082 28769542 PMC5518251

[B5] Cao S. J. (2016). *The Summarization of Hu Guo-jun’s Academic Thought and Experience on Diagnosing and Treating Depression and Clinical Research*. Doctoral dissertation, Nanjing University Of Chinese Medicine, Nanjing.

[B6] Chen C. K. Tsai Y. C. Hu H. J. Wu I. W. Sun C. Y. Chou C. C. (2010). Depression and suicide risk in hemodialysis patients with chronic renal failure. *Psychosomatics* 51:528. 10.1176/appi.psy.51.6.528 21051686

[B7] Chen H. M. You Z. L. (2005). Analysis e feature of regulating solution stasis in Fuqingzhu Medicine for women. *Guid. J. TCM.* 11 4–5.

[B8] Dorgalaleh A. Mahmudi M. Tabibian S. Khatib Z. K. Tamaddon G. H. Moghaddam E. S. (2013). Anemia and thrombocytopenia in acute and chronic renal failure. *Int. J. Hematol. Oncol. Stem Cell. Res.* 7 34–39. PMC3915422 24505541

[B9] Du J. Wang Y. Hunter R. (2009). Dynamic regulation of mitochondrial function by glucocorticoids. *Proc. Natl. Acad. Sci. U.S.A.* 106 3543–3548. 10.1073/pnas.0812671106 19202080 PMC2637276

[B10] Fatih D. Nevzat G. Faruk K. Ulu R. Atmaca M. (2016). The impact of red blood cell distribution width and neutrophil/lymphocyte ratio on the diagnosis of major depressive disorder. *Neurol. Ther.* 5 27–33. 10.1007/s40120-015-0039-8 26686339 PMC4919129

[B11] Fournier N. M. Duman R. S. (2012). Role of vascular endothelial growth factor in adult hippocampal neurogenesis: implications for the pathophysiology and treatment of depression. *Behav. Brain Res.* 227 440–449. 10.1016/j.bbr.2011.04.022 21536078 PMC3176958

[B12] Fraenkel P. G. (2017). Anemia of inflammation: a review. *Med. Clin. North Am.* 101 285–296. 10.1016/j.mcna.2016.09.005 28189171 PMC5308549

[B13] Gao L. Huang P. Dong Z. Gao T. Huang S. Zhou C. (2018). Modified Xiaoyaosan (MXYS) exerts anti-depressive effects by rectifying the brain blood oxygen level-dependent fMRI signals and improving hippocampal neurogenesis in mice. *Front. Pharmacol.* 9:1098. 10.3389/fphar.2018.01098 30323763 PMC6173122

[B14] Hunter R. G. Seligsohn M. Rubin T. G. Griffiths B. B. Ozdemir Y. Pfaff D. W. (2016). Stress and corticosteroids regulate rat hippocampal mitochondrial DNA gene expression via the glucocorticoid receptor. *Proc. Natl. Acad. Sci. U.S.A.* 113 9099–9104. 10.1073/pnas.1602185113 27457949 PMC4987818

[B15] Ji P. Wei Y. Hua Y. Zhang X. Yao W. Ma Q. (2018). A novel approach using metabolomics coupled with hematological and biochemical parameters to explain the enriching-blood effect and mechanism of unprocessed *Angelica sinensis* and its 4 kinds of processed products. *J. Ethnopharmacol.* 211 101–116. 10.1016/j.jep.2017.09.028 28958590

[B16] Joharapurkar A. A. Pandya V. B. Patel V. J. Desai R. C. Jain M. R. (2018). Prolyl hydroxylase inhibitors: a breakthrough in the therapy of anemia associated with chronic diseases. *J. Med. Chem.* 61 6964–6982. 10.1021/acs.jmedchem.7b01686 29712435

[B17] Li P. Yin Y. L. Li D. Kim S. W. Wu G. (2013). Amino acids and immune function. *Br. J. Nutr.* 98 237–252. 10.1017/S000711450769936X 17403271

[B18] Li P. L. Sun H. G. Hua Y. L. Ji P. Zhang L. Li J. X. (2015). Metabolomics study of hematopoietic function of *Angelica sinensis* on blood deficiency mice model. *J. Ethnopharmacol.* 166 261–269. 10.1016/j.jep.2015.03.010 25797116

[B19] Li W. Tang Y. Guo J. Shang E. Qian Y. Wang L. (2014). Comparative metabolomics analysis on hematopoietic functions of herb pair Gui-Xiong by ultra-high-performance liquid chromatography coupled to quadrupole time-of-flight mass spectrometry and pattern recognition approach. *J. Chromatogr. A* 1346 49–56. 10.1016/j.chroma.2014.04.042 24794940

[B20] Liu Y. Li H. Wu S. (2017). Antidepressant effects of water extracts from *Angelica sinensis* (Oliv) Diels. in mice. *Pharmacol. Clin. Chin. Mater. Med.* 33 106–108.

[B21] Mitrache C. Passweg J. Libura J. Petrikkos L. Seiler W. O. Gratwohl A. (2001). Anemia: an indicator for malnutrition in the elderly. *Ann. Hematol.* 80 295–298. 11446733 10.1007/s002770100287

[B22] Murakami M. Matsuno T. Ueda M. (1999). Stress polycythemia and organ choice(On the organ choice in psychosomatic disorders). *Japan. J. Psychosom. Med.* 39 145–152.

[B23] Newsholme P. Bender K. Kiely A. Brennan L. (2007). Amino acid metabolism, insulin secretion and diabetes. *Biochem. Soc. Trans.* 35 1180–1186. 17956307 10.1042/BST0351180

[B24] Østergaard L. Jørgensen M. B. Knudsen G. M. (2018). Low on energy? An energy supply-demand perspective on stress and depression. *Neurosci. Biobehav. Rev.* 94 248–270. 10.1016/j.neubiorev.2018.08.007 30145282

[B25] Pamuk G. E. Top M. Ş Uyan ık M. Ş K öker H. Akker M. Ak R. (2016). Is iron-deficiency anemia associated with migraine? Is there a role for anxiety and depression? *Wien Klin Wochenschr.* 128 576–580. 10.1007/s00508-015-0740-8 25854909

[B26] Pang H. Q. Yue S. J. Tang Y. P. Chen Y. Y. Tan Y. J. Cao Y. J. (2018). Integrated metabolomics and network pharmacology approach to explain possible action mechanisms of Xin-Sheng-Hua Granule for treating anemia. *Front. Pharmacol.* 9:165. 10.3389/fphar.2018.00165 29551975 PMC5840524

[B27] Penninx B. W. Pahor M. Cesari M. Corsi A. M. Woodman R. C. Bandinelli S. (2004). Anemia is associated with disability and decreased physical performance and muscle strength in the elderly. *J. Am. Geriatr. Soc.* 52 719–724. 15086651 10.1111/j.1532-5415.2004.52208.x

[B28] Pferschy-Wenzig E. M. Koskinen K. Moissl-Eichinger C. Bauer R. (2017). A combined LC-MS metabolomics- and 16S rRNA sequencing platform to assess interactions between herbal medicinal products and human gut bacteria in vitro: a pilot study on Willow Bark extract. *Front. Pharmacol.* 8:893. 10.3389/fphar.2017.00893 29326584 PMC5733343

[B29] Pickett J. L. Theberge D. C. Brown W. S. Schweitzer S. U. Nissenson A. R. (1999). Normalizing hematocrit in dialysis patients improves brain function. *Am. J. Kidney Dis.* 33 1122–1130. 10352201 10.1016/S0272-6386(99)70150-2

[B30] Quirk S. E. Williams L. J. O’Neil A. Pasco J. A. Jacka F. N. Housden S. (2013). The association between diet quality, dietary patterns and depression in adults: a systematic review. *BMC Psychiatry* 13:175. 10.1186/1471-244X-13-175 23802679 PMC3706241

[B31] Ren L. Chen G. (2017). Rapid antidepressant effects of Yueju: a new look at the function and mechanism of an old herbal medicine. *J. Ethnopharmacol.* 203 226–232. 10.1016/j.jep.2017.03.042 28347831

[B32] Shen J. Zhang J. Deng M. Liu Y. Hu Y. Zhang L. (2016). The antidepressant effect of *Angelica sinensis* extracts on chronic unpredictable mild stress-induced depression is mediated via the up-regulation of the BDNF signaling pathway in rats. *Evid. Based Complement. Alternat. Med.* 2016 1–8. 10.1155/2016/7434692 PMC5014956 27642354

[B33] Singh J. C. Kakalij R. M. Kshirsagar R. P. Kumar B. H. Komakula S. S. Diwan P. V. (2015). Cognitive effects of vanillic acid against streptozotocin-induced neurodegeneration in mice. *Pharm. Biol.* 53 630–636. 10.3109/13880209.2014.935866 25472801

[B34] Tacchi R. Ferrari A. Loche A. Bertolini A. (2008). Sucrose intake: increase in non-stressed rats and reduction in chronically stressed rats are both prevented by the gamma-hydroxybutyrate (GHB) analogue, GET73. *Pharmacol. Res.* 57 464–468. 10.1016/j.phrs.2008.05.004 18573666

[B35] Testai F. D. Kilkus J. P. Berdyshev E. Gorshkova I. Natarajan V. Dawson G. (2014). Multiple sphingolipid abnormalities following cerebral microendothelial hypoxia. *J. Neurochem.* 131 530–540. 10.1111/jnc.12836 25060904 PMC4349382

[B36] Urrechaga E. Hoffmann J. J. Izquierdo S. Escanero J. F. (2015). Differential diagnosis of microcytic anemia: the role of microcytic and hypochromic erythrocytes. *Int. J. Lab. Hematol.* 37 334–340. 10.1111/ijlh.12290 25181647

[B37] Vulser H. Wiernik E. Hoertel N. Thomas F. Pannier B. Czernichow S. (2016). Association between depression and anemia in otherwise healthy adults. *Acta Psychiatr. Scand.* 134 150–160. 10.1111/acps.12595 27238642

[B38] Wang J. Li X. He S. Hu L. Guo J. Huang X. (2018). Regulation of the kynurenine metabolism pathway by Xiaoyao San and the underlying effect in the hippocampus of the depressed rat. *J. Ethnopharmacol.* 214 13–21. 10.1016/j.jep.2017.11.037 29217494

[B39] Wang T. Sun H. G. Hua Y. L. (2016). Urine metabonomic study for blood- replenishing mechanism of Angelica sinensis in a blood-deficient mouse model. *Chin. J. Nat. Med.* 14 210–219. 10.1016/S1875-5364(16)30018-8 27025368

[B40] Wu F. Z. Xu W. J. Deng B. Liu S. D. Deng C. Wu M. Y. (2018). Wen-Luo-Tong decoction attenuates paclitaxel-induced peripheral neuropathy by regulating linoleic acid and glycerophospholipid metabolism pathways. *Front. Pharmacol.* 9:956. 10.3389/fphar.2018.00956 30233366 PMC6127630

[B41] Xu F. Peng D. Tao C. Yin D. Kou J. Zhu D. (2011). Anti-depression effects of Danggui-Shaoyao-San, a fixed combination of Traditional Chinese Medicine, on depression model in mice and rats. *Phytomedicine* 18 1130–1136. 10.1016/j.phymed.2011.05.002 21664113

[B42] Yang M. Dang R. Xu P. Guo Y. Han W. Liao D. (2018). Dl-3-n-Butylphthalide improves lipopolysaccharide-induced depressive- like behavior in rats: involvement of Nrf2 and NF-κB pathways. *Psychopharmacology* 235 2573–2585. 10.1007/s00213-018-4949-x 29943092

[B43] Zeni A. L. Zomkowski A. D. Maraschin M. Rodrigues A. L. Tasca C. I. (2012). Ferulic acid exerts antidepressant-like effect in the tail suspension test in mice: evidence for the involvement of the serotonergic system. *Eur. J. Pharmacol.* 679 68–74. 10.1016/j.ejphar.2011.12.041 22266492

[B44] Zhang G. (2013). *The Study of Zhu Danxi’s Academic Thinking of the Six Theory of Depression*. Doctoral dissertation. Heilongjiang university of Chinese medicine, Heilongjiang.

[B45] Zhang X. Li J. Xie B. Wu B. Lei S. Yao Y. (2018). Comparative metabolomics analysis of cervicitis in human patients and a phenol mucilage-induced rat model using liquid chromatography tandem mass spectrometry. *Front. Pharmacol.* 9:282. 10.3389/fphar.2018.00282 29670527 PMC5893906

[B46] Zhang Y. Ge J. F. Wang F. F. Liu F. Shi C. Li N. (2017). Crassifoside H improve the depressive-like behavior of rats under chronic unpredictable mild stress: possible involved mechanisms. *Brain Res. Bull.* 135 77–84. 10.1016/j.brainresbull.2017.09.015 28970041

[B47] Zhang Y. J. Huang X. Wang Y. Xie Y. Qiu X. J. Ren P. (2011). Ferulic acid-induced anti-depression and prokinetics similar to Chaihu-Shugan-San via polypharmacology. *Brain Res. Bull.* 86 222–228. 10.1016/j.brainresbull.2011.07.002 21791239

[B48] Zhou K. Jia N. Jiang N. Wang F. Kou J. (2015). Beneficial effect of Danggui-Shaoyao-San, a traditional Chinese medicine, on drowsiness induced by chronic restraint stress. *Neurosci. Lett.* 597 26–31. 10.1016/j.neulet.2015.04.030 25907199

[B49] Zhou Y. Lu L. Li Z. Gao X. Tian J. Zhang L. (2011). Antidepressant-like effects of the fractions of Xiaoyaosan on rat model of chronic unpredictable mild stress. *J. Ethnopharmacol.* 137 236–244. 10.1016/j.jep.2011.05.016 21640181

[B50] Zhu S. Guo S. Duan J. A. Qian D. Yan H. Sha X. (2017). UHPLC-TQ-MS coupled with multivariate statistical analysis to characterize nucleosides, nucleobases and amino acids in angelicae sinensis radix obtained by different drying methods. *Molecules* 22:E918. 10.3390/molecules22060918 28587175 PMC6152706

